# 4-step, 2-h carboplatin desensitization in Japanese patients with ovarian cancer: a prospective study

**DOI:** 10.1007/s10147-021-01935-7

**Published:** 2021-05-26

**Authors:** Meiko Nishimura, Hideki Sakai, Takuma Onoe, Shogen Boku, Takaaki Yokoyama, Genmu Kadokura, Satoshi Morita, Noriyuki Katsumata, Koji Matsumoto

**Affiliations:** 1 Department of Medical Oncology, Hyogo Cancer Center, 13-70, Kitaoji-cho, Akashi, Hyogo 673-8558 Japan; 2 Department of Medical Oncology, Nippon Medical School Musashi Kosugi Hospital, Kanagawa, Japan; 3 Department of Biomedical Statistics and Bioinformatics, Kyoto University Graduate School of Medicine, Kyoto, Japan; 4 Present Address: Cancer Treatment Center, Kansai Medical University Hospital, Osaka, Japan; 5 Present Address: Department of Medical Oncology, Suwa Central Hospital, Nagano, Japan

**Keywords:** Carboplatin desensitization, Ovarian cancer, Hypersensitivity reactions

## Abstract

**Background:**

Carboplatin is a key drug for ovarian cancer. However, it sometimes induces hypersensitivity reactions (HSRs) that result in the discontinuation of the treatment. Although various desensitization protocols have been reported in previous retrospective studies, a limited number of prospective studies have analyzed these protocols.

**Methods:**

Patients with platinum-sensitive relapsed ovarian cancer who experienced carboplatin-induced HSRs were treated with diluted solutions of 1/1000, 1/100, 1/10 and an undiluted solution of carboplatin over a 1-h period. If no HSRs occurred within the first two cycles, a short protocol regimen over a 30-min period per solution was followed. The primary endpoint was treatment completion rate.

**Results:**

Between May 2015 and September 2018, 21 patients were enrolled from two institutions. One patient experienced platinum-sensitive recurrence after the desensitization protocol; thus, 22 sessions were analyzed. Epinephrine use, treatment-related death, and intensive care unit (ICU) admissions did not occur. The median number of desensitization cycles was 6 (range 1–6). Two sessions were discontinued early because of grade 2 dysgeusia and grade 2 malaise. Treatment in two (9.1%) patients was discontinued because of HSR development. The treatment completion rate was 90.9%. Six (27.3%) sessions met the criteria for transition to the short protocol regimen. In 14 (63.6%) sessions, HSRs were observed during infusion of the undiluted solution. The median progression-free survival and overall survival were 14.8 and 23.8 months, respectively.

**Conclusion:**

This 4-step, 2-h carboplatin desensitization protocol is safe and feasible. Patients require careful monitoring with a rapid response to HSRs, especially during the administration of undiluted solutions.

**Supplementary Information:**

The online version contains supplementary material available at 10.1007/s10147-021-01935-7.

## Introduction

Despite initial treatment with surgical induction and chemotherapy by carboplatin plus paclitaxel, the majority of patients with ovarian cancer relapse. Platinum-sensitive ovarian cancer is defined as a platinum-free interval of 6 months or longer. Treatment for platinum-sensitive recurrent ovarian cancer typically includes a carboplatin-containing regimen, such as carboplatin plus paclitaxel, gemcitabine, pegylated liposomal doxorubicin [1–3], and carboplatin plus paclitaxel or gemcitabine or pegylated liposomal doxorubicin with bevacizumab [4–6]. The median survival time of carboplatin rechallenge in patients with platinum-sensitive recurrent ovarian cancer is approximately 30 months. Carboplatin is a key drug, especially for platinum-sensitive recurrent ovarian cancer. However, it sometimes induces hypersensitivity reactions (HSRs) that result in the discontinuation of treatment. Although platinum-sensitive, the fact that some patients cannot tolerate carboplatin because of HSRs has been the greatest disadvantage of this treatment.

The incidence of HSRs in patients who received more than seven cycles of carboplatin was greater than 27%, and half of those HSRs were moderate to severe [7]. Carboplatin rechallenge in patients who have experienced carboplatin-induced HSRs can potentially induce life-threatening HSRs. Although several desensitization protocols have been reported from various institutions, the majority of these protocols are retrospective studies performed by a single institution [8–10]. Furthermore, and despite the fact that they have a 12-step methodology, these prospective protocols are complicated [11, 12]. For example, the speedup of each of the three solutions used (1/100, 1/10, and undiluted) is a 4-step process, with each step lasting for 15 min [11, 12]. Initial desensitization for these 12-step protocols is performed in an intensive care unit (ICU) [11, 12]. Instead, and to simplify and improve generalizability, we developed a 4-step, 2-h protocol, which resulted in an 80% completion rate [9]. In a previous study, three out of four breakthrough HSRs occurred in the first or second cycle [9]. Consequently, we speculated that it was feasible to reduce the dripping time (from 60 to 30 min per solution) to further improve generalizability.

To our knowledge, there are no prospective studies that have analyzed the feasibility of 4-step, 2-h carboplatin desensitization. In addition, there are no studies that have reported on breakthrough HSRs in detail. Therefore, it is essential to assess and define specific information that could efficiently standardize the desensitization process, such as the number of cycles, the solutions that are appropriate for this process, the time of onset of breakthrough HSRs, and the time required prior to restarting desensitization.

In this study, we conducted a multi-institution prospective study to evaluate the safety, efficacy, and specific details of breakthrough HSRs of a 4-step, 2-h carboplatin desensitization protocol.

## Patients and methods

### Patients

Patients (≥ 20 years old) with platinum-sensitive recurrent ovarian cancer who had experienced carboplatin-induced HSRs were included in this study. Carboplatin-induced HSRs were defined by clinical judgment; adverse events greater than grade 1 using Common Terminology Criteria for Adverse Events (CTCAE), version 4.0, related to carboplatin allergy during administration of carboplatin (Supplementary Table 1). Additional eligibility criteria included an Eastern Cooperative Oncology Group performance status of 0–2, and adequate hematologic, renal, and hepatic functions. Patients with habitual use of antihistamines or systemic steroids were excluded, as were patients with concurrent severe complications (poor control of diabetes or hypertension), active systemic infections, or double cancer with a prognosis of less than 1 year.

### Study design and treatment

Premedication of each regimen was not fixed in our protocol, and showed in it is presented as a Supplementary Table 2. Carboplatin AUC and regimen interval were also not fixed in our protocol and were performed according to standard treatment. Patients were treated with diluted solutions of 1/1000, 1/100, 1/10 and an undiluted solution of carboplatin over a 1-h period. HSRs were defined as adverse events in accordance with the allergic reactions that had occurred since the beginning of this process and until immediately after carboplatin desensitization. If adverse events greater than grade 3 and diagnosed as HSRs were observed, treatment was discontinued. Epinephrine was recommended for grade 3 or greater HSRs. If grade 1 or 2 HSRs were observed, treatment was interrupted; when recovered to grade 0, treatment restart was permitted. If recurrence of a grade 1 or 2 HSR was observed, the former procedure was again followed, and for HSRs that did not recover to grade 0 within 1 h, the protocol treatment was stopped. However, it was also necessary to wait for a period of more than 1 h to evaluate whether HSRs that did not completely recover to grade 0 showed any improvement. If no HSRs occurred within the first two cycles, a short protocol regimen was allowed over a 30-min period per solution. We allowed patients who transitioned to the short regimen to become outpatients. The treatment continued until the number of conventional cycles was reached or until disease progression. This protocol was performed by well-trained staff that were rapidly responsive to HSRs and who were supervised by board-certified medical oncologists in the general ward (not in the ICU). This study was approved by the institutional review boards of all the participating organizations and was registered with the UMIN clinical trials registry (000016603). All patients provided written informed consent.

### Assessments

Treatment completion rate was the primary endpoint of this study. We defined completion rate as the proportion of patients who started and completed the desensitization protocol. Patients who discontinued the desensitization protocol, either because of the development of adverse events other than HSRs or because of the development of a progressive disease, were included in the completion population. Secondary endpoints included the response rate, potential toxicities, the number of treatments, the rate of switch to the short regimen, progression-free survival (PFS), and the overall survival (OS). Adverse events including allergic reactions related to carboplatin were assessed using the CTCAE, version 4.0. We defined initial HSRs as the HSRs occurring before enrollment in this desensitization study, and breakthrough HSRs as the HSRs occurring throughout this desensitization study.

### Statistical analysis

Previous desensitization studies showed a completion rate that was over 80%. Consequently, we defined a threshold of 75% completion rate to be an indicator of the desensitization protocol’s effectiveness. Furthermore, the expectation rate in previous studies ranged between 90 and 100% [6–12]; thus, we established an expectation of 95% completion rate. We planned for 80% power and an *α* error of 5%, and we assumed a sample size of 20 participants.

## Results

Between May 2015 and September 2018, 21 patients were enrolled from two institutions. One patient experienced platinum-sensitive recurrence after the desensitization protocol; thus, 22 sessions were analyzed in our study. One session translates into one sequence of treatments, for example, six cycles of desensitization. The median age was 57 years (range 41–76), and the most common histological type was serous adenocarcinoma (63.6%) (Table 1). Regimen was not changed in cases where the initial HSRs occurred during cycles, and we permitted patients with platinum-sensitive recurrence to change regimen in cases where the initial HSRs had occurred in the previous last cycles. The median cumulative number of carboplatin cycles at initial HSR was 10 (range 4–22), and 90.9% of the patients received more than seven cycles of carboplatin. The most common initial HSRs were urticaria, dyspnea, hypotension, and pruritus (Table 2).

**Table 1:** Patients characteristics

Characteristics	
Age, years	
Median (range)	57 (41–76)
ECOG PS—no. (%)	
0	19 (86.4)
1	3 (13.6)
Stage—no. (%)	
IIIB	3 (13.6)
IIIC	10 (45.5)
IV	9 (40.9)
Histological type—no. (%)	
Serous adenocarcinoma	14 (63.6)
Adenocarcinoma	4 (18.2)
High-grade serous carcinoma	2 (9.1)
Clear cell carcinoma	2 (9.1)
Prior lines of platinum regimen	
Median (range)	2 (1–3)
Prior cycles of carboplatin—no. (%)	
< 7	2 (9.1)
≧ 7	20 (90.9)
Regimen during this treatment—no. (%)	
CBDCA + PLD	7 (31.8)
CBDCA + GEM	6 (27.3)
CBDCA + DTX	4 (18.2)
CBDCA + PTX	3 (13.6)
CBDCA + GEM + BEV	1 (4.5)
CBDCA monotherapy	1 (4.5)

*ECOG PS* Eastern Cooperative Oncology Group performance status, *CBDCA* carboplatin, *PLD* pegylated liposomal doxorubicin, *GEM* gemcitabine, *DTX* docetaxel, *PTX* paclitaxel, *BEV* bevacizumab

**Table 2:** Initial HSRs

HSRs	*N* (%)			
	Grade 1	Grade 2	Grade 3	Total
Anaphylaxis	0	0	1 (4.5)	1 (4.5)
Allergic reaction	1 (4.5)	4 (18.2)	0	5 (22.7)
Hypotension	4 (18.2)	1 (4.5)	2 (9.1)	7 (31.8)
Urticaria	8 (36.4)	1 (4.5)	0	9 (40.9)
Pruritus	3 (13.6)	3 (13.6)	0	6 (27.3)
Erythema	0	2 (9.1)	0	2 (9.1)
Dyspnea	6 (27.3)	1 (4.5)	2 (9.1)	8 (36.4)
Laryngopharyngeal dysesthesia	1 (4.5)	0	0	1 (4.5)
Diarrhea	0	1 (4.5)	0	1 (4.5)
Abdominal pain	0	1 (4.5)	0	1 (4.5)
Vomiting	1 (4.5)	1 (4.5)	0	2 (9.1)
Nausea	1 (4.5)	0	0	1 (4.5)

**Fig. 1:**
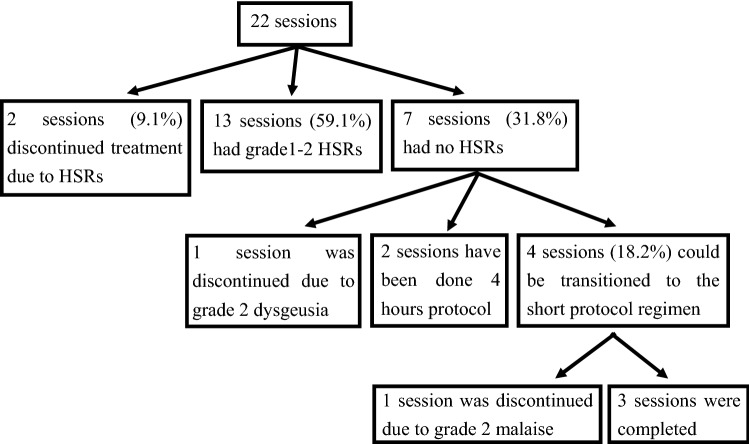
Desensitization delivery

Desensitization delivery is shown in Fig. 1. Seven (31.8%) sessions revealed no HSRs, whereas six (27.3%) sessions met the criteria of transition to the short protocol regimen, and four (18.2%) sessions were transitioned to the short protocol regimen. Two (9.1%) sessions were discontinued early because of adverse events other than HSRs: grade 2 dysgeusia at Cycle 1 and grade 2 malaise at Cycle 5. The treatment completion rate, which was the primary endpoint, was 90.9%.

**Fig. 2:**
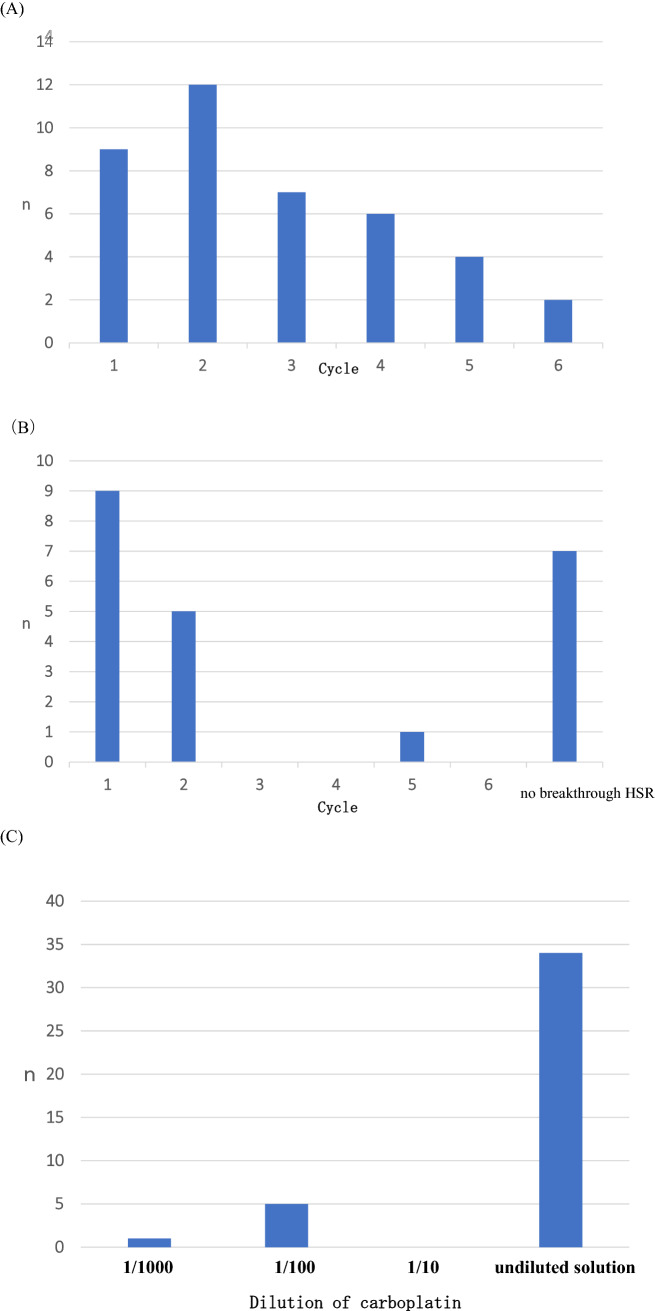

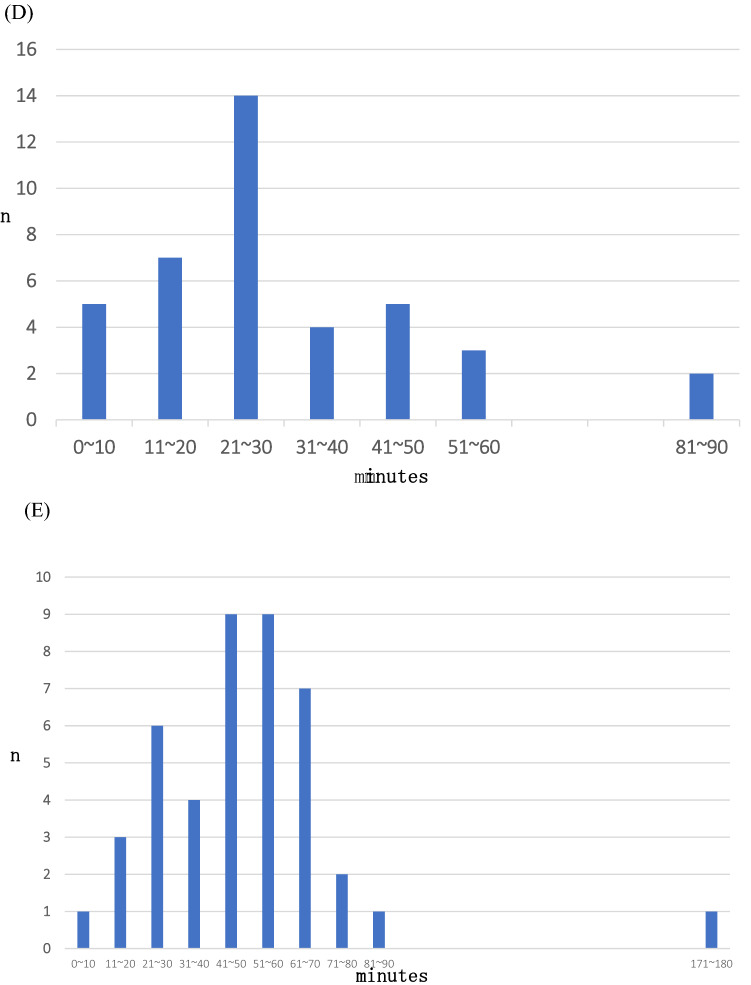
Details of breakthrough HSRs. **A** Timing of the cycle number at breakthrough HSRs at every cycle. The total number refers to HSR events. **B** Timing of the cycle number at breakthrough HSRs at every session. The total number refers to the number of sessions. **C** Concentration of carboplatin at breakthrough HSRs at every cycle. **D** Timing from the start of every solution to the development of breakthrough HSRs at every cycle. **E** Time from interruption to protocol restart. Total number of patients was 43 because patients who restarted their therapy after interruption were also included

Fifteen (68.2%) sessions revealed a certain degree of breakthrough HSRs, and the details of the breakthrough HSRs are summarized in Fig. 2. Most cycle number timings at breakthrough HSR were 1 and 2, which comprised 52.5% at every cycle and 63.6% at every session (Fig. 2A, B). Breakthrough HSRs occurred with undiluted solution in 85% of cases (Fig. 2C). Thirty-five percent of all timings from the beginning of every solution until breakthrough HSRs ranged from 21 to 30 min (Fig. 2D). Furthermore, most timings from interruption point until restart of the protocol ranged between 40 and 60 min (41.9%) (Fig. 2E). Among the sessions with breakthrough HSRs, 13 (59.1%) sessions had grade 1–2 HSRs (Fig. 1). Two (9.1%) patients had to discontinue their treatment owing to breakthrough HSRs: a patient with grade 3 hypotension due to fecal incontinence managed to recover immediately after the provision of hydration and corticosteroids, and a patient with grade 3 urticaria that lasted for 3 h (Fig. 1). The median number of desensitization cycles was 6 (range 1–6). Breakthrough HSRs that occurred throughout the delivery of our protocol are shown in Table 3, and the most common breakthrough HSRs involved urticaria (grade 1: 36.4%, grade 2: 9.1%, grade 3: 4.5%). In 14 (63.6%) sessions, breakthrough HSRs were observed during infusion of the undiluted solution. Grade 4 HSRs, treatment-related deaths, and ICU admissions did not occur.

**Table 3:** Breakthrough HSRs

HSRs	*N* (%)			
	Grade 1	Grade 2	Grade 3	Total
Allergic reaction	0	0	1 (4.5)*	1 (4.5)
Hypotension	0	0	1 (4.5)*	1 (4.5)
Urticaria	8 (36.4)	2 (9.1)	1 (4.5)	11 (49.5)
Pruritus	0	4 (18.2)	0	4 (18.2)
Erythema	0	2 (9.1)	0	2 (9.1)
Dyspnea	3 (13.6)	1 (4.5)	1 (4.5)	5 (22.7)
Hypoxia	0	0	1 (4.5)	1 (4.5)
Laryngopharyngeal dysesthesia	2 (9.1)	1 (4.5)	0	3 (13.5)
Cough	1 (4.5)	0	0	1 (4.5)
Diarrhea	0	0	1 (4.5)*	1 (4.5)
Abdominal pain	0	0	1 (4.5)*	1 (4.5)
Vomiting	0	1 (4.5)	0	1 (4.5)
Sinus tachycardia	0	1 (4.5)	0	1 (4.5)
Back pain	1 (4.5)	0	0	1 (4.5)

*Same patients

The median PFS and the median OS were 14.8 months (range 2.0–46.2) and 23.8 months (range 2.2–59.0), respectively. The response rate was 63.6%, and the disease control rate was 100%.

## Discussion

This carboplatin desensitization study is the first prospective study to evaluate a 2-h desensitization protocol for Japanese cancer patients and provide a detailed description of breakthrough HSRs. Four (18.2%) sessions could be transitioned to the short protocol regimen over a 30-min period per solution. We provided these patients with the opportunity to shift to the short regimen in an outpatient basis. However, because of their refusal, all patients included in this study were treated in a general unit and there was no ICU admission. The various methods and completion rates in previous carboplatin desensitization studies are shown in Table 4 [8–14]. The completion rate in our study (90.9%) met its primary endpoint and was the highest among studies that do not allow the use of epinephrine.

**Table 4:** Previous carboplatin desensitization studies

	*N*	Initial dilution degree	Step	Hour	Completion rate (%)	Epinephrine use	Author
Retrospective	33	1/1000	4	16.5	88	No	Rose et al. [8]
	20	1/1000	4	4	80	No	Takase et al. [9]
	129	1/1000	4	3.5	96	–	Gary et al. [10]
	48	Undiluted	4	1.5	96	Yes	Vetter et al. [13]
		Undiluted	16	4.25			
		1/100	16	9			
Prospective	23	1/1000	4	6	95	–	Confino-cohen et al. [14]
	44	1/100	12	5.8	100	Yes	Lee et al. [11]
				3.8			
	60	1/100	12	5.8	100	Yes	Castells et al. [12]
	21	1/1000	4	2	90.9	No	This study

Recently, one-solution protocols have been reported including carboplatin. Vetter et al*.* have reported a 96.6% completion rate of their retrospective one-solution desensitization protocol [13]. However, the respective infusion rates of the standard (16-step) desensitization protocol were complicated [13]. In a prospective study of a one-solution protocol, infusion rates were also complicated and changed every 15 min [15]. Two patients had grade 3 HSRs in the carboplatin subgroup, and one patient required epinephrine. However, in contrast to our study, there were no detailed recordings of the exact respective timings [15].

Breakthrough HSRs occurred in seven sessions, of which six sessions were completed after interruption and the use of a histamine blocker or steroid. Only one session failed to recover to grade 0 within 1 h and was, therefore, discontinued in accordance with our protocol. In our study, most sessions were completed, even in cases where breakthrough HSRs occurred. In previous studies, the completion rate in which epinephrine use was allowed was 96–100% [11–13]. In contrast, the completion rates in studies with no epinephrine use were below 90% [8, 9]. In our protocol, epinephrine use was recommended when grade 3 HSRs occurred. However, our study did not include any sessions that used epinephrine because the only patient case with grade 3 hypotension as a result of fecal incontinence managed to recover immediately after the provision of hydration and corticosteroids. Had we used epinephrine earlier, a certain number of the discontinued sessions would have been completed. As a result, we recommend the proactive use of epinephrine to improve the completion rate.

The regimens of seven sessions with no breakthrough HSRs were all carboplatin and pegylated liposomal doxorubicin. However, the association between the use of drugs and carboplatin remains unclear owing to the small number of subjects included. The occurrence rates of both hypotension and dyspnea in initial HSRs (31.8% and 36.4%) decreased in breakthrough HSRs (4.5% and 22.7%, respectively). In contrast, the incidence rate of urticaria was increased (from 40.9 to 49.5%). Consequently, carboplatin desensitization may reduce the development of severe breakthrough HSR, such as hypotension or dyspnea.

This desensitization protocol was safely performed in a non-ICU setting with a well-trained team that was supervised by board-certified medical oncologists. When using this protocol, medical staff needs to be prepared for a rapid response to HSRs, because severe HSRs can induce life-threatening conditions and thus terminate the subsequent treatment. We recommend that each solution should take at least 30 min, while carefully monitoring each solution halfway, because HSRs may often occur between 21 and 30 min since the beginning of every solution. Development of HSRs with undiluted solution occurred in 63.6% of all sessions. Therefore, it is necessary to ensure more careful monitoring and rapid response to HSRs during this period. Our study reveals that development of HSRs after two cycles was a rare incidence, provided that there were no HSRs observed during these two cycles. After two cycles without breakthrough HSRs, a less intense method might be feasible, such as reducing the infusion time or moving desensitization at an ambulatory therapy center.

In the INOVATYON study, the median PFS and OS of carboplatin plus polyethylene glycol-coated liposomal doxorubicin in patients with platinum-sensitive relapsed ovarian cancer were 9.0 and 21.3 months, respectively [16]. Furthermore, the median PFS and OS of patients treated with platinum-based chemotherapy (with the majority of patients receiving carboplatin plus paclitaxel) in the MITO-8 study were 9.0 and 24.5 months, respectively [17]. Similarly, the median PFS and OS values in our carboplatin desensitization study (PFS 14.8 months, OS 23.8 months) achieved comparable efficacy with standard platinum combination chemotherapy for platinum-sensitive ovarian cancer [16, 17]. Moreover, the use of PARP inhibitors as a maintenance therapy in patients with platinum-sensitive exhibited a significantly improved PFS in relapsed ovarian cancer [18, 19]. Therefore, it can be inferred that carboplatin desensitization played an important role in patients with platinum-sensitive ovarian cancer experiencing HSR, because patients must have received at least four cycles of platinum doublets in both pivotal trials. In our study, only two patients received PARP inhibitors because this drug has only been recently approved (2018) for maintenance therapy in patients with platinum-sensitive ovarian cancer in Japan.

Several reports on BRCA1/2 mutations that are associated to HSRs of carboplatin have been published [20, 21]. However, our study did not include patients with BRCA1/2 mutations because approval for the investigation of such cases was only recently granted in Japan.

Our study has several limitations. First, a relatively small sample size is included. In addition, the number of patients that could be transitioned to the short protocol regimen was small, i.e., four sessions (18.2%), and there were no outpatients included. Therefore, further investigations are warranted to verify our findings.

In conclusion, the carboplatin desensitization protocol described in this study is safe and feasible. Patients receiving this protocol require careful monitoring and rapid response to HSRs, especially during the administration of undiluted solutions, because more than half of the total HSRs and all severe HSRs that resulted in treatment termination occurred during this period.

## Supplementary Information

Below is the link to the electronic supplementary material.

Supplementary file1 (DOCX 17 kb)

Supplementary file2 (DOCX 15 kb)
