# Supplementary material for: 4-step, 2-h carboplatin desensitization in Japanese patients with ovarian cancer: a prospective study
Source: Int J Clin Oncol. 2021 May 26;26(8):1553–60. doi: 10.1007/s10147-021-01935-7 (PMC8286943; doi:10.1007/s10147-021-01935-7)
Supplement: Supplementary file 1 — Supplementary file1 (DOCX 17 kb) [file 10147_2021_1935_MOESM1_ESM.docx]

**Supplemental Table 1**

|  |  | Grade1 | Grade2 | Grade3 | Grade4 |
| --- | --- | --- | --- | --- | --- |
| Respiratory disorders | Dyspnea | Shortness of breath with moderate exertion | Shortness of breath with minimal exertion; limiting instrumental ADL | Shortness of breath at rest; limiting self care ADL | Life-threatening consequences; urgent intervention indicated |
|  | Wheezing | Detectable airway noise with minimal symptoms | Moderate symptoms; medical intervention indicated; limiting instrumental ADL | Severe respiratory symptoms limiting self care ADL; oxygen therapy or hospitalization indicated | Life-threatening consequences; urgent intervention indicated |
|  | Hypoxia | - | Decreased oxygen saturation with exercise (e.g., pulse oximeter <88%); intermittent supplemental oxygen | Decreased oxygen saturation at rest (e.g., pulse oximeter <88% or PaO2 <=55 mm Hg) | Life-threatening airway compromise; urgent intervention indicated (e.g., tracheotomy or intubation) |
| Cardiac disorders | Hypotension | Asymptomatic, intervention not indicated | Non-urgent medical intervention indicated | Medical intervention or hospitalization indicated | Life-threatening and urgent intervention indicated |
|  | Sinus bradycardia | Asymptomatic, intervention not indicated | Symptomatic, medical intervention indicated | Severe, medically significant, medical intervention indicated | Life-threatening consequences; urgent intervention indicated |
|  | Sinus tachycardia | Asymptomatic, intervention not indicated | Symptomatic, medical intervention indicated | Urgent medical intervention indicated | - |
| Skin disorders | Urticaria | Urticarial lesions covering <10% BSA; topical intervention indicated | Urticarial lesions covering 10 - 30% BSA; oral intervention indicated | Urticarial lesions covering >30% BSA; IV intervention indicated | - |
|  | Pruritus | Mild or localized; topical intervention indicated | Intense or widespread; intermittent; skin changes from scratching (e.g., edema, papulation, excoriations, lichenification, oozing/crusts); oral intervention indicated; limiting instrumental ADL | Intense or widespread; constant; limiting self care ADL or sleep; oral corticosteroid or immunosuppressive therapy indicated | - |
| Gastrointestinal disorders | Nausea | Loss of appetite without alteration in eating habits | Oral intake decreased without significant weight loss, dehydration or malnutrition | Inadequate oral caloric or fluid intake; tube feeding, TPN, or hospitalization indicated | - |
|  | Vomiting | 1 - 2 episodes (separated by 5 minutes) in 24 hrs | 3 - 5 episodes (separated by 5 minutes) in 24 hrs | >=6 episodes (separated by 5 minutes) in 24 hrs; tube feeding, TPN or hospitalization indicated | Life-threatening consequences; urgent intervention indicated |
|  | Diarrhea | Increase of <4 stools per day over baseline; mild increase in ostomy output compared to baseline | Increase of 4 - 6 stools per day over baseline; moderate increase in ostomy output compared to baseline | Increase of >=7 stools per day over baseline; incontinence; hospitalization indicated; severe increase in ostomy output compared to baseline; limiting self care ADL | Life-threatening consequences; urgent intervention indicated |
| Nervous system disorders | Depressed level of consciousness | Decreased level of alertness | Sedation; slow response to stimuli; limiting instrumental ADL | Difficult to arouse | Life-threatening consequences |
|  | Dizziness | Mild unsteadiness or sensation of movement | Moderate unsteadiness or sensation of movement; limiting instrumental ADL | Severe unsteadiness or sensation of movement; limiting self care ADL | - |
|  | Seizure | Brief partial seizure; no loss of consciousness | Brief generalized seizure | Multiple seizures despite medical intervention | Life-threatening; prolonged repetitive seizures |
| Immune system disorders | Allergic reaction | Transient flushing or rash, drug fever <38 degrees C (<100.4 degrees F); intervention not indicated | Intervention or infusion interruption indicated; responds promptly to symptomatic treatment (e.g., antihistamines, NSAIDS, narcotics); prophylactic medications indicated for <=24 hrs | Prolonged (e.g., not rapidly responsive to symptomatic medication and/or brief interruption of infusion); recurrence of symptoms following initial improvement; hospitalization indicated for clinical sequelae (e.g., renal impairment, pulmonary infiltrates) | Life-threatening consequences; urgent intervention indicated |
|  | Anaphylaxis | - | - | Symptomatic bronchospasm, with or without urticaria; parenteral intervention indicated; allergy-related edema/angioedema; hypotension | Life-threatening consequences; urgent intervention indicated |
