# Supplementary material for: 4-step, 2-h carboplatin desensitization in Japanese patients with ovarian cancer: a prospective study
Source: Int J Clin Oncol. 2021 May 26;26(8):1553–60. doi: 10.1007/s10147-021-01935-7 (PMC8286943; doi:10.1007/s10147-021-01935-7)
Supplement: Supplementary file 2 — Supplementary file2 (DOCX 15 kb) [file 10147_2021_1935_MOESM2_ESM.docx]

**Supplemental Table 2.** Premedication regimens

| Regimen | Our institution | The other institution |
| --- | --- | --- |
| CBDCA + PLD | Dex 12mg + 5-HT3 RA + NK1 RA | Dex 8mg + 5-HT3 RA |
| CBDCA + GEM | Dex 12mg + 5-HT3 RA + NK1 RA | Dex 8mg + 5-HT3 RA |
| CBDCA + DTX | Dex 12mg + 5-HT3 RA + NK1 RA + H1 blocker + H2 blocker | Dex 12mg + 5-HT3 RA |
| CBDCA + PTX | Dex 12mg + 5-HT3 RA + NK1 RA + H1 blocker + H2 blocker | Dex 8mg + 5-HT3 RA + H2 blocker |
| CBDCA + GEM +BEV |  | Dex 8mg + 5-HT3 RA |
| CBDCA monotherapy |  | Dex 8mg + 5-HT3 RA |

**Abbreviations:** Dex, Dexamethasone; 5-HT3 RA, 5-HT3 receptor antagonist; NK1 RA, NK1 receptor antagonist; CBDCA, carboplatin; PLD, pegylated liposomal doxorubicin; GEM, gemcitabine; DTX, docetaxel; PTX, paclitaxel; BEV, bevacizumab.
